# Access to clinical trials among oncology patients: results of a cross sectional survey

**DOI:** 10.1186/s12885-017-3644-3

**Published:** 2017-09-18

**Authors:** Mariko Carey, Allison W. Boyes, Rochelle Smits, Jamie Bryant, Amy Waller, Ian Olver

**Affiliations:** 1 Priority Research Centre for Health Behaviour, School of Medicine & Public Health, Faculty of Health, University of Newcastle, W4, HMRI Building, Callaghan, NSW 2308 Australia; 2 Hunter Medical Research Institute, New Lambton, NSW Australia; 3 Samson Institute for Health Research, University of South Australia, Adelaide, South Australia Australia

**Keywords:** Patient participation, Controlled clinical trials, Randomized, Neoplasms, Cancer, Informed consent, Patient education

## Abstract

**Background:**

Clinical trials are necessary for the advancement of cancer treatment and care, however low rates of participation in such trials limit the generalisability of findings. The objective of this study was to examine the proportion of medical oncology outpatients in Australia who are invited and consent to participate in clinical trials and the factors associated with this.

**Methods:**

A sample of adult medical oncology patients was recruited from three Australian cancer treatment centres. Consenting patients completed two paper-and-pencil surveys; one at the time of consent and another approximately 1 month later. A multivariate logistic regression was conducted to explore factors associated with invitation and participation in a trial.

**Results:**

Thirty-eight percent (*n* = 146) of the 383 participants reported they had been invited to take part in a clinical trial. Of those invited, 93% reported consenting to participate in the trial, with the majority indicating that they did not regret their decision (89%). Treatment centre and time since diagnosis were significantly associated with being invited to take part in a clinical trial. None of the factors examined were associated with clinical trial consent rates.

**Conclusions:**

The main barrier to clinical trial participation is not being invited to do so, with the centre the patient attends being a modifiable determinant of whether or not they are invited. Increasing the resources available to treatment centres to ensure all patients are offered participation in trials they are eligible for may help to improve rates of trial participation.

**Electronic supplementary material:**

The online version of this article (10.1186/s12885-017-3644-3) contains supplementary material, which is available to authorized users.

## Background

Randomised clinical trials provide the strongest evidence about whether a new treatment is better than an existing treatment. While clinical trials are characterised by high internal validity, they are often criticised as providing poor evidence of external validity [1]. In order to maximise generalisability, high participant enrolment rates must be achieved and the recruited sample must reflect the diversity of the population to which the results will be applied. However, only 2–3% of adult cancer patients in the United States [2] and between 2 [3] and 11% [4] of those in Australia are reported to participate in clinical trials. This has led to cancer control organisations reorganising clinical research infrastructure, setting targets and allocating dedicated resources in an effort to increase trial participation [3, 5].

Several groups based on age, race, geographic location, sex and socioeconomic status are under-represented in cancer clinical trials. For example, while two thirds of cancer patients are elderly, only 22–30% of clinical trial participants are aged 65 years or over [6, 7]. Racial and ethnic minority groups including African Americans, Hispanics and Asian/Pacific Islanders are also less likely to enrol in clinical trials [8]. Rural patients are significantly less likely to be recruited than their urban counterparts [9]. Men with colorectal cancer and lung cancer are more likely than women with these diseases to participate in clinical trials [7]. Socioeconomic barriers are also evident, with individuals of lower income and lower education less likely to participate in clinical trials [10].

Clinical trials regularly close prior to meeting their recruitment target, or take significantly longer than expected [11]. Low enrolment rates into clinical trials are the result of a complex array of factors that operate at the patient, clinician and systems levels [12]. Strict eligibility criteria in clinical trials may make it less likely that certain groups of patients may be eligible [13]. For example, people with co-morbid conditions are often excluded from participation, contributing to low enrolment rates among elderly people [14, 15]. Clinician attitudes [16], including concerns related to the ethics of randomisation [17], resource constraints such as insufficient staff or physical resources [4], and perceived patient burden [17] may influence willingness to enrol patients in trials.

Organisational factors also play a role, with distance between a clinician’s practice and the nearest clinical trial centre inversely related to clinicians’ recruitment rates to trials [18]. Not all hospitals are clinical trials active. Non-academic medical centres also have lower patient recruitment rates due to limited clinical trials infrastructure, workforce and diversity [5]. For trials targeting uncommon cancers, patient availability may pose challenges for achieving an adequate sample size [4].

Much of the evidence on biases in trial participation among cancer patients has been derived in the United States. It is not clear the extent to which these findings are applicable to other countries. Australia has a universal health care system in which the government provides free treatment at public hospitals, and subsidises the cost of some prescription medicines. Therefore, financial barriers to accessing treatment, and hence trials [10], may be reduced in comparison to the USA context.

The aims of this study were to examine, among a sample of Australian medical oncology patients: 1) the proportion who are invited to and agree to participate in a clinical trial; 2) factors associated with a) being invited to participate, and b) consenting to participate in a clinical trial; 3) reasons for non-participation among those who report not consenting to clinical trials; and 4) views about who should determine whether patients are approached to participate in multiple trials.

## Methods

### Setting

The current study was conducted as part of a larger cross-sectional study examining psychological outcomes among medical oncology patients. This study was conducted in three cancer treatment centres in Australia. Data were collected between November 2012 and August 2014. All treatment centres were located in public teaching hospitals with links to Universities and had a clinical trials unit. Treatment centres A and C were located in capital cities, while treatment centre B was located in a major regional area. Treatment centres A and B had 200–500 beds; while treatment centre C had more than 500 beds. Ethics approvals were obtained from the Human Research Ethics Committees of the University of Newcastle, Cancer Institute of New South Wales, as well as institutional ethics committees.

### Participants

Patients who were aged 18 or older, diagnosed with cancer, English speaking and presenting for a medical oncology outpatient appointment were eligible to participate. Those attending the medical oncology clinic for the first time, and those unable to provide informed consent due to cognitive impairment or mental illness were excluded.

### Procedure

A research assistant approached eligible patients in the clinic to seek written informed consent. Consenting patients were asked to complete a paper and pencil survey either in clinic or at home. Those who elected to take the survey home were asked to return it within a week in the reply paid envelope provided. This survey included questions about sociodemographic, disease and treatment variables, as well as questions about psychological wellbeing. Approximately 1 month later, a second survey was mailed to participants. The questions on clinical trial participation reported here were included in the second survey. Up to two reminder letters were sent to non-responders after 3 and 6 weeks.

### Measures

#### Sociodemographic characteristics

Self-report data was collected on age, sex, highest level of education, Aboriginal and/ or Torres Strait Islander status, marital status, country of birth, home post code, living situation, employment status, private health insurance status, concession card status, and smoking status. In Australia, concession cards are issued by the government to low income earners to allow access to cheaper health services and medications. Private health insurance provides cover to patients to be treated as a private patient in a public or private hospital, by the doctor of their choice.

#### Disease and treatment variables

The following data were also collected by self-report: cancer type, perceived stage of disease at diagnosis (early versus advanced), perceived remission status, time since diagnosis, current treatments (e.g surgery, chemotherapy etc), and main reason for hospital visit on the day of recruitment (e.g. to receive treatment, check-up after completing treatment, etc).

#### Participation in clinical trials

Participants were provided with the following definition of a clinical trial to aid them in answering the questions: “A clinical trial is a research study where participants are assigned by chance (randomly) to receive the new treatment or usual treatment.” Participants were asked whether, since their cancer diagnosis, they had been invited to take part in a clinical trial (yes/ no). Those who responded “yes” were asked to indicate what the trial was about using the following response options: “surgical treatments”, “radiation therapy”, “chemotherapy”, “complementary therapy”, “psychological well-being”, “can’t remember”, or “other”. More than one response could be selected. Respondents were then asked whether they had agreed to participate in the trial (yes/ no).

#### Views regarding trial participation

Those who had agreed to participate in a trial were asked whether they would make the same choice again: “Now that you think back, would you agree to take part in the trial again?” (yes/no/not sure). Those who indicated that they had declined to participate were asked to indicate their reasons for non-participation from the following options: “I do not like the idea of clinical trials”, “I wanted to choose my treatment”, “I did not understand what was involved”, “I was worried about risks/side effects”, “my loved ones did not want me to”, and “other”.

#### Views regarding participation in multiple trials

Participants were given the following instructions: “Imagine that you are participating in a clinical trial and a new trial comes up that you could participate in as well. What should happen?” Response options included not being asked about the second trial; the researcher checking with the patient’s doctor first, and the patient being asked directly if they wanted to participate. A copy of the items assessing participation and views regarding clinical trials is available as an Additional file 1.

### Statistical analysis

Frequencies and percentages with 95% confidence intervals were calculated for all variables of interest. Characteristics of the sample (gender, cancer type and age) were compared to national data using a one sample chi-square test. Fisher’s exact test was used to explore factors associated with being asked to participate in a trial. Those with a *p*-value <0.1 were included in a multivariate exact logistic regression. Odds ratios (95% confidence intervals) and exact *p*-values were calculated. All statistical analyses were programmed using SAS v9.4 [19].

## Results

Of the 968 patients screened for eligibility, 179 (18%) were ineligible. Of the 789 eligible patients, 605 (77%) consented to take part in the study. Characteristics of the 504 consenters who provided information on age and sex were compared to non-consenters. There was a higher proportion of females among consenters (χ^2^ = 18.1, df = 2, *p* = 0.0001) and slightly higher proportions of those aged 65+ for non-consenters (χ^2^ = 12.6, df = 6, *p* = 0.0488). Three hundred and eighty-three patients (63%) completed both the baseline survey and clinical trials questions and were included in the analyses. One hundred and fifty-two (40%) of these participants were recruited from treatment centre A; 111 (29%) from treatment centre B, and 120 (31%) from treatment centre C.

Demographic and disease characteristics of the sample are presented in Table 1. Comparison with national cancer incidence data [20] indicated that females were over-represented in the current sample (χ^2^ = 48.28, df = 1, *p* < .0001). The distribution of cancer types was also significantly different to the national incidence data (χ^2^ = 352.41, df = 5, p < .0001), with the current sample having a greater proportion of breast and colorectal cancer patients and a lower proportion of prostate and melanoma patients. There were no differences in the proportion of those aged 65 and older between the current sample and national data (χ^2^ = 0.04, df = 1, *p* > .05).

**Table 1:** Sociodemographic, disease and treatment characteristics for participants who had and had not been invited to take part in a clinical trial

		Have you been asked to take part in a clinical trial?		
Variable	Category	Yes (*n* = 146)	No (*n* = 237)	Chi-square *p*-value
Centre	1	74 (49%)	78 (51%)	0.0005
	2	28 (25%)	83 (75%)	
	3	44 (37%)	76 (63%)	
Age	Less than 55	36 (34%)	69 (66%)	0.2089
	55 to 74	90 (41%)	127 (59%)	
	75 and over	18 (31%)	41 (69%)	
Gender	Male	59 (41%)	84 (59%)	0.3290
	Female	87 (36%)	153 (64%)	
Aboriginal and/ or Torres Strait Islander	Non indigenous	140 (37%)	234 (63%)	0.3701*
	Indigenous	3 (60%)	2 (40%)	
Marital	Married/living with partner	92 (38%)	147 (62%)	0.7937
	Single/divorced/separated/widowed	52 (37%)	88 (63%)	
Education	High school or less	76 (39%)	117 (61%)	0.5317
	University or vocational	63 (36%)	111 (64%)	
Country of birth	Australia	105 (39%)	167 (61%)	0.6075
	Other	39 (36%)	70 (64%)	
Health insured	Yes	65 (44%)	82 (56%)	0.0332
	No	77 (33%)	154 (67%)	
Concession card	Yes	86 (37%)	147 (63%)	0.7000
	No	56 (39%)	88 (61%)	
Smoking status	Current smoker	16 (37%)	27 (63%)	0.9513
	Former smoker	65 (37%)	110 (63%)	
	Never smoked	62 (39%)	98 (61%)	
Location of residence	City/inner regional	119 (38%)	192 (62%)	0.9282
	Outer regional/remote	26 (38%)	43 (62%)	
Living arrangements	With others	110 (37%)	184 (63%)	0.7784
	Alone	34 (39%)	53 (61%)	
Employment	Full-time	26 (42%)	36 (58%)	0.1934
	Part-time	15 (27%)	40 (73%)	
	All others	103 (39%)	159 (61%)	
Time since diagnosis	12 m or less	51 (31%)	115 (69%)	0.0029
	13 to 24 m	38 (54%)	32 (46%)	
	Over 24 m	57 (40%)	86 (60%)	
Surgery	No	39 (41%)	55 (59%)	0.4808
	Yes	107 (37%)	179 (63%)	
Chemotherapy	No	25 (28%)	64 (72%)	0.0286
	Yes	118 (41%)	170 (59%)	
Radiotherapy	No	45 (34%)	88 (66%)	0.4610
	Yes	83 (38%)	137 (62%)	
Hormone therapy	No	92 (37%)	157 (63%)	0.6777
	Yes	36 (35%)	68 (65%)	
Biological therapy	No	107 (34%)	205 (66%)	0.0341
	Yes	21 (51%)	20 (49%)	
Cancer stage at diagnosis	Early	58 (32%)	124 (68%)	0.4376
	Not applicable / Do not know	9 (45%)	11 (55%)	
	Progressed/advanced	21 (37%)	36 (63%)	
Remission:	Do not know	20 (29%)	48 (71%)	0.0306
	In remission	24 (30%)	57 (70%)	
	Not in remission	66 (44%)	83 (56%)	
Cancer type	Haematological/blood cancer	5 (63%)	3 (38%)	0.3362
	Breast	52 (33%)	108 (68%)	
	Colorectal	21 (38%)	35 (63%)	
	Prostate	11 (33%)	22 (67%)	
	Lung	13 (42%)	18 (58%)	
	Melanoma	7 (50%)	7 (50%)	
	More than one type or other	33 (45%)	41 (55%)	
Reason for visit: combined	To discuss treatment	20 (37%)	34 (63%)	0.9153
	To receive treatment/check-up during treatment	60 (38%)	99 (62%)	
	Check-up after treatment/other	65 (40%)	99 (60%)	

Note: Numbers for each category may not sum to 383 due to missing data. Percentages may not sum to 100 due to rounding

*p-value from exact tests

### Rates of clinical trial invitation by trial type

One hundred and forty six (38%; 95% CI = 33.23–43.0) respondents reported that they had been invited to take part in a clinical trial. The number of respondents who had been invited by type of trial is shown in Table 2. Eighteen (13%; 95% CI = 7.2–18.3) respondents reported being invited to take part in more than one type of trial, with the most common combination being radiation therapy and chemotherapy trials (*n* = 7).

**Table 2:** Number and percentage of participants invited to take part in a clinical trial by trial type

Trial type	Total (n = 146)	% (95% CI)
Surgical treatments	5	3.4 (0.4–6.4)
Radiation therapy	21	14 (8.6–20.1)
Chemotherapy treatments	73	50 (41.8–58.2)
Complementary or natural therapies	6	4.1 (0.8–7.4)
Psychological wellbeing	9	6.1 (2.2–10.1)
Cannot remember	17	11.6 (6.4–16.9)
Other	30	20.5 (13.9–27.2)

### Rates of participation and reasons for non-participation

Of those who were invited to take part in a trial, 129 (93%; 95% CI =88.4–97.1) reported that they had consented. That is, overall, 33% (95% CI =28.0–37.3) of the sample reported having participated in clinical trial.

For the 17 respondents (11%; 95% CI =6.2–16.6) who were invited but declined participation, ten provided reasons for non-participation: ‘other’ reasons (*n* = 5) which included accessibility, feeling too unwell to participate, feeling that too much else was going on, and already being involved in a trial; wanting to choose own treatment (*n* = 2); concern regarding risk factors and side effects from participation (n = 2), and not liking the idea of clinical trials (*n* = 1).

Of those who reported that they had agreed to participate, 112 respondents (89%; 95% CI = 83.3–94.4) said that if asked to make the decision again that they would still choose to participate.

### Characteristics associated with being invited and consenting to participate in a clinical trial

The univariate analysis showed that treatment centre, private health insurance, time since diagnosis, remission, chemotherapy and biological treatment were associated with being asked to take part in a clinical trial (*p* < 0.1). None of the factors examined were associated with the decision to take part in a clinical trial (data not shown). After adjusting for multiple variables, there was a significant association between treatment centre, time since diagnosis and being asked to take part in a clinical trial. Participants from treatment centre B had increased odds of being asked to participate in a trial compared to those as centre A. The odds of being asked to participate in a trial decreased with increasing time since diagnosis. All other associations were found to be non-significant after adjusting for confounders (Table 3).

**Table 3:** Factors associated with being asked to take part in a clinical trial as the outcome

Predictor	Comparison	OR (95% CI)	Type 3 p-value
Centre	B vs A	2.90 (1.27, 6.61)	0.0386
	C vs A	1.93 (0.93, 4.01)	
Private Health Insurance	Yes vs No	0.73 (0.41, 1.29)	0.2759
Time Since Diagnosis	13 to 24 months vs 12 months or less	0.24 (0.11, 0.53)	0.0010
	Over 24 months vs 12 months or less	0.46 (0.25, 0.87)	
Chemotherapy treatment	Yes vs No	0.65 (0.34, 1.24)	0.1873
Biological treatment	Yes vs No	0.61 (0.27, 1.37)	0.2295
Remission	Not in remission vs In remission	0.60 (0.30, 1.22)	0.2321
	Do not know vs In remission	0.52 (0.23, 1.21)	

### Views on being approached to participate in multiple trials

The majority of respondents (*n* = 91; 68%; 95% CI =59.9–75.9) agreed that “I should be asked directly if I want to participate in the second trial, and given the option to discuss it with my doctor if I want to.” Forty respondents (30%; 95% CI =22.0–37.7) indicated “the researcher should check if my doctor thinks I should participate before discussing it with me”, while only 3 (2%; 95% CI =0.0–4.8) respondents said they “should not be asked about the second trial”.

### Discussion

Overall 33% of the sample reported having participated in a clinical trial. This rate is very high compared to other studies which have reported rates of participation in the range of 3 to 18% [4, 10, 21]. This may reflect that our sample over-represented women with breast cancer and people with colorectal cancer. A recent review found representation of breast cancer in clinical trials is proportionate to its incidence, whereas lung cancer has the highest incidence and highest mortality (27.6%), but accounts for only 9.2% of trials [22].

The overwhelming majority of those asked to participate in a trial reported that they had agreed to do so (93%). This rate is higher than other studies that have reported rates of acceptance into clinical cancer trials of 51–72% [21, 23]. Further, most reported that they would make the same decision to participate in a clinical trial again (89%). Together these results suggest that the biggest barrier to participation in a trial is not being invited to do so. Due to the study methods, however, it was not possible to determine the underlying reasons for not being invited to take part in a clinical trial. For example, we do not know whether trials were available at the treatment centres respondents attended, nor the eligibility of respondents for those trials. However, previous studies show that even after patients are deemed eligible by physicians and appropriate clinical trials for the cancer type and stage of disease are available, 40–50% of cancer patients will still not be offered participation in a trial [21].

Reasons reported for declining participation in a trial were broadly consistent with past research. For example, similar to our study, other research has indicated that being overwhelmed, unwell, and preferring to choose one’s own treatment rather than be randomised to a treatment condition [15, 21, 23–25] as well as fear of side effects [24] act as key barriers to participation.

Those diagnosed over 12 months ago were significantly less likely to be invited to take part in a trial, with the odds of being invited to participate decreasing with increasing time since diagnosis. This may reflect that more clinical trials are likely to be available for those who are newly diagnosed. Therefore, as time since diagnosis increases, recall of invitations to participate in trials during the early post-diagnosis period may decrease. The higher proportion of recently diagnosed people who reported being asked to take part in trials may also reflect the impact of national initiatives to increase trial participation; and increasing recognition of the critical role that clinical trials play in evidence-based health care.

Treatment centre was the only other factor found to be associated with being invited to participate in a trial in the multivariable analysis. Those attending centre B had 2.79 greater odds of having been invited to participate than those at treatment centre A. These results are surprising given that treatment centre B was located in a regional area rather than a large metropolitan area, where greater research infrastructure and hence trial activity might be expected. While only a small number of treatment centres participated in the current study, results are consistent with previous reports suggesting considerable variation in trial activity between centres [5]. Previous research has reported that the number of oncologists and the presence of an approved multidisciplinary cancer program were significantly associated with higher accrual to clinical trials [26], however, we did not measure these characteristics.

In contrast to past research [21, 26–31], we found no evidence that age, socioeconomic factors, or country of birth were associated with being invited to participate in a trial. It is possible that the lack of an association between country of birth and trial invitation was due to the exclusion of non-English speakers from the sample. There was also no association between cancer type and trial invitation. This is at odds with other Australian research which has identified a disproportionate number of trials on breast cancer [32] and the relative rarity of haematological cancer trials [4].

### Implications

The treatment centre was a major determinant of being asked to take part in a clinical trial. High acceptability of trials was indicated by high reported consent rates and reported willingness to be offered the opportunity to participate in more than one trial. Together these findings suggest that one way to increase clinical trial participation rates may be to upskill and/ or resource hospitals with low rates of trial activity so that they can actively recruit for a range of clinical trials. This may have the advantage of ensuring equity of access to trials for a broader range of patients as well as enabling more rapid recruitment of the trial sample. However, if such measures are to be effectively implemented it is likely that some simplification to the complex multi-site ethics processes that researchers face may be necessary. The need to obtain multiple approvals and comply with a range of organisation-specific requirements may act as a significant barrier to expanding trial activity to a broader range of institutions.

### Limitations

The generalisability of our findings may be limited due to the small number of centres that participated in our study, and over-representation of women and those with breast cancer. It is also possible that non-consenters to clinical trials were under-represented in the sample. Further, translation of survey instruments into languages other than English and provision of interpreter services were cost prohibitive. Therefore, we had to exclude people who were not fluent in English from participating. Lack of representation of culturally and linguistically diverse populations within our sample may have resulted in the prevalence of being approached to participate in clinical trials being overestimated. Given these limitations, further investigation of our findings in a larger and more diverse sample of treatment centres and patients is warranted.

Data regarding trial participation was self-reported by respondents. While a clear definition was provided about what participation in a clinical trial constituted, it is possible that participants did not accurately recall these discussions with their healthcare providers.

Data on clinical and treatment variables were based on patient self-report due to the prohibitive cost of extracting this information from hospital medical records. One study comparing the accuracy of self-reported disease and treatment among 895 women with breast cancer to medical records data, showed that for general questions about whether the person had had surgery, chemotherapy, radiotherapy or hormone treatment), accuracy was very high (100%, 99%, 99 and 94% respectively) [33]. Agreement about whether a recurrence had occurred was also high at 90%; while agreement regarding stage at diagnosis was lower at 60%. This suggests that most self-reported data about basic cancer disease and treatment information is likely to be accurate.

Finally, although few cancer patients receive all of their cancer care at one treatment centre, we did not assess whether participants were invited to and participated in a trial at the treatment centre from which they were recruited. The inclusion of this information in future surveys may help to clarify the role that treatment centre plays in clinical trial access.

## Conclusions

The biggest modifiable determinant of access to a clinical a trial is the treatment centre. Higher rates of accrual to clinical cancer trials may be achieved by increasing the resources available to healthcare providers to ensure all eligible patients are offered participation in appropriate trials.

## Additional file

Additional file 1: Clinical Trials Questionnaire. Participation in Clinical Trials: Questionnaire items assessing participation in clinical trials, views regarding trial participation, and views regarding participation in multiple trials. (PDF 267 kb)

## Abbreviations

CI: Confidence interval
DF: Degrees of freedom
USA: United States of America
