# Supplementary material for: Access to clinical trials among oncology patients: results of a cross sectional survey
Source: BMC Cancer. 2017 Sep 18;17:653. doi: 10.1186/s12885-017-3644-3 (PMC5604159; doi:10.1186/s12885-017-3644-3)
Supplement: Additional file 1: — Clinical Trials Questionnaire. Participation in Clinical Trials: Questionnaire items assessing participation in clinical trials, views regarding trial participation, and views regarding participation in multiple trials. (PDF 267 kb) [file 12885_2017_3644_MOESM1_ESM.pdf]

Participation in Clinical Trials

This next section asks about your experiences with taking part in clinical trials. A clinical trial is a research study where participants are assigned by chance (randomly) to receive the new treatment or usual treatment. Please circle the number that best describes your answer.

B1. **Since your cancer diagnosis, have you been asked to take part in a clinical trial?**

1 Yes
2 No *(go to Section C on the next page)*

B2. **What was the clinical trial about?**

Please circle all that apply

1 Surgical treatments
2 Radiation therapy treatments
3 Chemotherapy treatments
4 Complementary or natural therapies
5 Psychological well being
6 Can't remember
7 Other (please specify)______________________

B3. **Did you choose to participate in the clinical trial?**

(If you have been asked to participate in more than one trial, please think about the last trial you were invited to take part in when answering)

1 Yes
2 No *(go to Question 5)*

B4. **Now that you think back, would you agree to take part in the clinical trial again?**

Please circle one number only

1 Yes *(go to Question 6)*
2 No *(go to Question 6)*
3 Not sure *(go to Question 6)*

B5 **Why didn't you want to participate in the trial?**

Please circle one number only

1 I do not like the idea of clinical trials
2 I wanted to choose my treatment
3 I did not understand what it involved
4 I was worried about the risks/side effects
5 My loved ones did not want me to
6 Other (please specify____________________________

B6. **Imagine that you are participating in a clinical trial and a new trial comes up that you could participate in as well. What should happen?**

Please circle one number only

1 I should not be asked about the second trial
2 The researcher should check if my doctor thinks I should participate in the trial before discussing it with me
3 I should be asked directly if I want to participate in the second trial, and given the option to discuss it with my doctor if I want to

---
